# Cardiac EndMT and EpiMT as a developmental continuum: integration of mechanical, metabolic, and epigenetic regulation

**DOI:** 10.3389/fcell.2026.1892888

**Published:** 2026-08-14

**Authors:** Diwen Li, Shijun Hu, Tianli Zhao

**Affiliations:** 1 Department of Cardiovascular Surgery, The Second Xiangya Hospital, Central South University, Changsha, China; 2 Department of Cardiovascular Surgery, Second Affiliated Hospital of Zhejiang University, Hangzhou, China; 3 Clinical Center for Gene Diagnosis and Therapy, The Second Xiangya Hospital of Central South University, Changsha, Hunan, China

**Keywords:** cardiac EMT, congenital heart disease, EndMT, EpiMT, mechanotransduction, single-cell multi-omics

## Abstract

Cardiac epithelial–mesenchymal transition (EMT) is a conserved morphogenetic program that shapes embryonic heart development and contributes to disease when aberrantly reactivated. Two major forms of cardiac EMT, endocardial-to-mesenchymal transition (EndMT) and epicardial epithelial-to-mesenchymal transition (EpiMT), support valve and septal formation, outflow tract remodeling, coronary vascular maturation, myocardial growth, and cardiac fibroblast generation. Increasing evidence indicates that these processes are not binary cell fate switches, but dynamic and context-dependent continua governed by coordinated signaling, mechanical, metabolic, extracellular matrix, and epigenetic inputs. In this review, we synthesize classical and emerging mechanisms regulating EndMT and EpiMT, including TGF-β/BMP, Notch, ErbB, Wnt/β-catenin, FGF, YAP/TAZ, chromatin remodeling, and non-coding RNA networks. We propose that developmental EndMT is best understood as a regionally licensed continuum of endocardial cell states in the atrioventricular canal and outflow tract, whereas developmental EpiMT represents a niche-dependent lineage continuum shaped by epicardial competence, myocardial-derived cues, mechanical environment, and post-EMT lineage allocation. This framework helps explain why similar regulatory pathways can generate distinct morphogenetic, fibrotic, or reparative outcomes depending on developmental stage, anatomical context, and cellular state. We further discuss how aberrant EndMT and EpiMT contribute to congenital heart disease, including valve malformations, septal defects, outflow tract defects, coronary abnormalities, and ventricular hypoplasia. Finally, we evaluate emerging approaches, including single-cell multi-omics, spatial transcriptomics, lineage tracing, live imaging, patient-specific induced pluripotent stem cell models, organoids, and human-based new alternative methodologies, as tools for resolving EMT heterogeneity, testing causal mechanisms, and defining therapeutic safety boundaries. Understanding cardiac EndMT and EpiMT as evidence-aware, context-dependent developmental continua may refine the interpretation of congenital and adult cardiac disease and inform future studies evaluating whether pathological EMT-related plasticity can be selectively modulated without impairing repair or vascular homeostasis.

## 1 Introduction

The vertebrate heart is the first functional organ to form during embryogenesis and relies on highly coordinated morphogenetic programs to achieve its precise architecture across species (Zaffran and Frasch, 2002). During the transition from a linear heart tube to a fully septated four-chambered organ, epithelial–mesenchymal transition (EMT) serves as a conserved and fundamental process driving cardiac morphogenesis (von Gise and Pu, 2012). Dysregulated EMT during development contributes to congenital heart defects, whereas aberrant reactivation of EMT or EMT-like programs in the adult heart has been implicated in cardiac fibrosis, valvular disease, and impaired repair after myocardial injury, underscoring the context-dependent roles of EMT in cardiac health and disease (von Gise and Pu, 2012; Wang et al., 2023). Intriguingly, EMT-mediated valve and septal formation exhibits striking parallels between mammals and lower vertebrates, such as zebrafish, suggesting that cardiac EMT is governed by an ancient genetic toolkit refined over approximately 500 million years of evolution (Bakkers, 2011). Nevertheless, subtle interspecies differences in EMT regulation, including the distinct roles of TGF-β isoforms in avian and mammalian models, highlight unresolved questions regarding the plasticity and context dependency of this process (Camenisch et al., 2002). This review focuses on two crucial yet distinct forms of cardiac EMT: endocardial-to-mesenchymal transition (EndMT), which is essential for valve and septal formation, and epicardial epithelial-to-mesenchymal transition (EpiMT), which contributes to coronary vascular development and cardiac fibroblast lineages (von Gise and Pu, 2012). Rather than treating EndMT and EpiMT as uniform binary conversions, we frame them as context-dependent transitional programs in which endocardial and epicardial cells may acquire partial EMT features, migratory capacity, and lineage-specific identities to varying degrees. The outcome of these transitions is shaped by developmental timing, anatomical location, mechanical and metabolic conditions, extracellular matrix composition, and paracrine signaling. This framework helps explain why similar regulatory pathways, including TGF-β, Notch, Wnt, BMP/FGF, ErbB, and YAP/TAZ, can support endocardial cushion formation, epicardial invasion, coronary vascular maturation, fibrotic remodeling, or limited reparative activation in different biological contexts. Accordingly, this review integrates classical and emerging regulatory mechanisms while distinguishing direct cardiac developmental evidence from findings inferred from disease, vascular, fibrotic, or non-cardiac models. By doing so, we aim to move beyond a pathway-by-pathway summary and provide a stage- and niche-dependent view of how EndMT and EpiMT contribute to cardiac morphogenesis, pathology, and repair.

Within this continuum framework, EMT-related states are defined by combinations of molecular, morphological, behavioral, and lineage features rather than by single markers. EMT is generally characterized by reduced apicobasal polarity, remodeling or disassembly of cell–cell junctions, cytoskeletal reorganization, and acquisition of migratory capacity (von Gise and Pu, 2012). EMT-primed cells largely retain epithelial or endothelial organization but show increased responsiveness to EMT-permissive cues, including early activation of EMT-associated transcriptional regulators, altered regulatory responsiveness, or weakening of junctional programs. Partial EMT refers to intermediate and often dynamic states in which epithelial/endothelial and mesenchymal features coexist; these cells show reduced polarity, weakened cell–cell junctions, and limited migratory behavior while retaining features of their epithelial or endothelial origin. Invasive mesenchyme describes cells with stronger migratory and matrix-invasive properties that have delaminated from the epithelial or endothelial layer and entered the cushion mesenchyme or the subepicardial/myocardial compartment. Post-EMT lineage allocation refers to the subsequent specification of EMT-derived cells into defined derivatives, such as cushion mesenchymal cells, cardiac fibroblasts, vascular smooth muscle cells, or pericytes (von Gise and Pu, 2012).

## 2 Developmental EndMT: region-specific licensing rather than universal pathway activation

**FIGURE 1:**
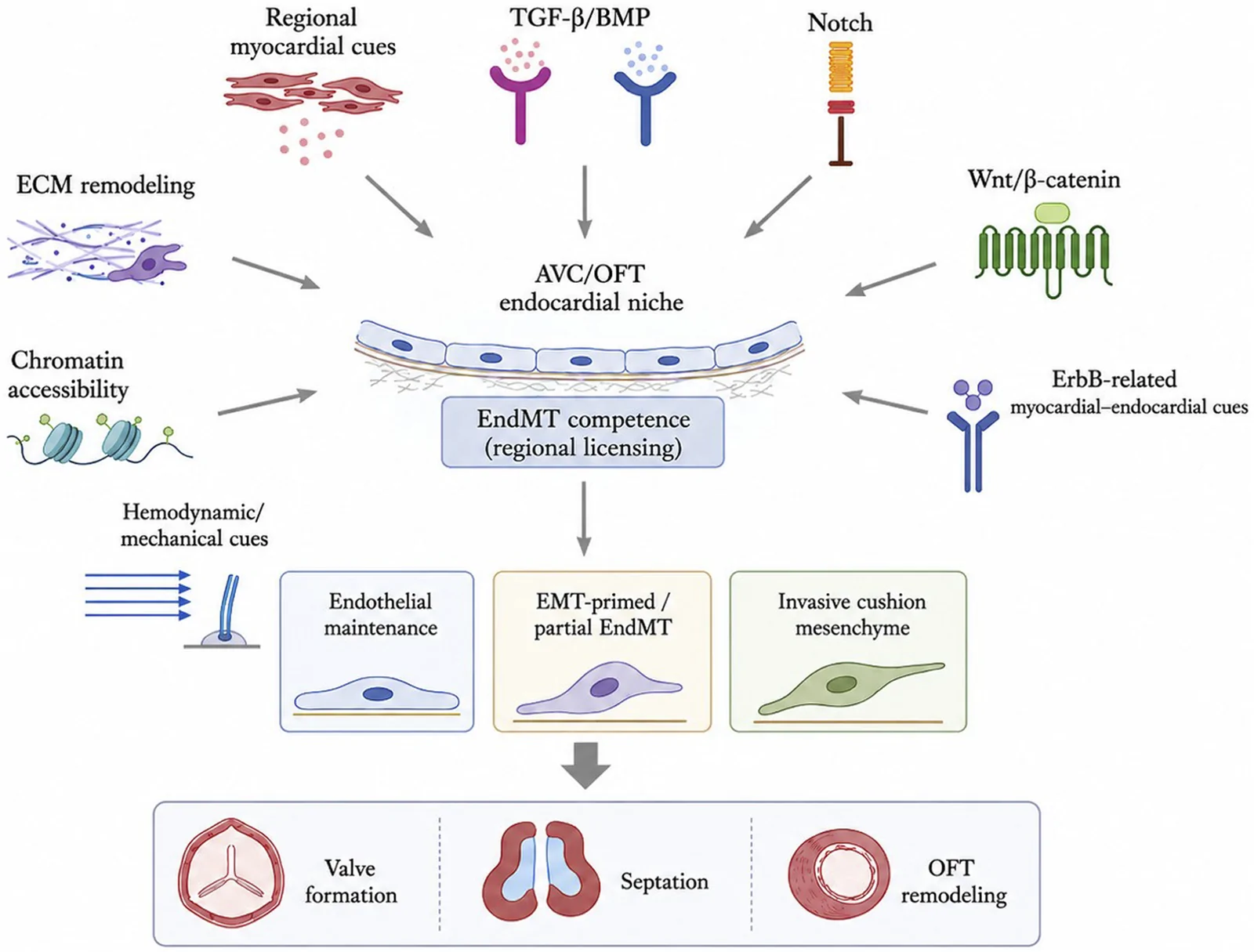
Regional licensing model of developmental EndMT. Developmental EndMT is illustrated as a regionally licensed continuum of endocardial cell states rather than a uniform response to individual pathway activation. In the atrioventricular canal (AVC) and outflow tract (OFT), EndMT competence emerges when multiple permissive inputs converge, including regional myocardial cues, TGF-β/BMP, Notch, Wnt/β-catenin, ErbB-related myocardial–endocardial cues, extracellular matrix remodeling, chromatin accessibility, and local hemodynamic or mechanical forces. Depending on developmental stage, anatomical niche, and cellular competence, endocardial cells may remain endothelial, enter EMT-primed or partial EndMT states, or progress toward invasive cushion mesenchyme. These coordinated state transitions support valve formation, septation, and OFT remodeling. EndMT, endocardial-to-mesenchymal transition; AVC, atrioventricular canal; OFT, outflow tract; TGF-β, transforming growth factor-β; BMP, bone morphogenetic protein; ErbB, erythroblastic oncogene B receptor family; ECM, extracellular matrix.

Developmental EndMT is a highly regionalized morphogenetic process rather than a uniform response of the entire endocardium to pro-EMT signals (Figure 1). During early heart development, EndMT is initiated within a restricted temporal window that coincides with cardiac looping and early chamber patterning (Liu and Nakano, 2024; Lotto et al., 2023). In avian embryos, this process begins around Hamburger–Hamilton stage 17 and continues through HH19, whereas in mice it starts at approximately embryonic day 9.5 (Inai et al., 2013; Chang et al., 2014). At these stages, EndMT is concentrated mainly in two anatomical domains: the atrioventricular canal (AVC) and the outflow tract (OFT). Endocardial cells in these regions acquire mesenchymal features, invade the cardiac jelly, and generate cushion mesenchyme that later contributes to valve and septal structures (Liu and Nakano, 2024; Berg et al., 2025). The spatial confinement of this process raises an important conceptual question: why do only selected endocardial populations undergo EndMT while adjacent chamber endocardium maintains endothelial identity?

A useful interpretation—developed throughout this review—is that developmental EndMT requires regional licensing: the progressive acquisition of mesenchymal features is permitted only when endocardial cells integrate regionally restricted myocardial signals (Ma et al., 2005; Luna-Zurita et al., 2010), extracellular matrix composition (Kern et al., 2006), hemodynamic forces (Berg et al., 2025), and permissive chromatin states (Akerberg et al., 2015). In this model, canonical signaling pathways do not function as isolated or universally sufficient EMT triggers. Instead, they establish or reinforce EndMT competence only in endocardial cells exposed to the appropriate combination of myocardial signals, extracellular matrix cues, hemodynamic forces, and chromatin accessibility (Luna-Zurita et al., 2010). Thus, the AVC and OFT should not be viewed merely as anatomical sites where EndMT happens, but as permissive niches in which molecular, mechanical, and extracellular inputs converge to allow selected endocardial cells to enter EMT-primed and invasive mesenchymal states.

### 2.1 TGF-β/BMP signaling establishes EndMT competence in a permissive niche

The TGF-β superfamily, especially BMP2/4 and TGF-β2/3, remains one of the best-supported regulatory axes in developmental EndMT. In the AVC, myocardial BMP2 promotes EndMT by activating BMP receptor signaling, inducing Snai1, repressing epithelial/endothelial adhesion programs, and reinforcing AVC identity through downstream factors such as Tbx2 (Ma et al., 2005; Wang et al., 2005; Song et al., 2007). TGF-β2 further supports cushion formation, with genetic and functional studies showing that loss or blockade of TGF-β2 impairs valve development and EndMT progression (Camenisch et al., 2002; Bartram et al., 2001). Downstream SMAD signaling cooperates with cardiac transcription factors such as GATA4 to activate EndMT-associated genes, including Has2, thereby linking transcriptional remodeling with the production of a hyaluronan-rich extracellular matrix required for cushion expansion (Shirai et al., 2009; Rajagopal et al., 2007).

However, the role of TGF-β/BMP signaling is better understood as the establishment of EndMT competence rather than simple induction of a universal mesenchymal program. BMP2 expression is regionally enriched in the AVC myocardium, and its downstream effects depend on local myocardial–endocardial interactions (Prados et al., 2018). In chamber myocardium, counter-regulatory mechanisms, including Hey1/2- and HDAC-associated repression of AVC-specific enhancers, help prevent inappropriate EndMT activation (Stefanovic et al., 2014; Fischer et al., 2007). This indicates that the same broad signaling family can produce different outputs depending on regional transcriptional and chromatin context (Stefanovic et al., 2014). Therefore, TGF-β/BMP signaling acts within a permissive developmental landscape: it helps selected endocardial cells become responsive to EMT induction, but it does not by itself explain the spatial precision of EndMT. This interpretation is also important when comparing developmental EndMT with disease-associated endothelial plasticity. TGF-β signaling can promote mesenchymal or fibrogenic programs in adult vascular and fibrotic settings, but these pathological responses should not be directly equated with embryonic cushion EndMT (Ma et al., 2020; Savorani et al., 2021). In development, TGF-β/BMP signaling contributes to organized tissue construction; in disease, related downstream modules may stabilize maladaptive mesenchymal or myofibroblast-like states (Lee and Massagué, 2022; Bastos et al., 2025). The difference lies not only in ligand activity, but also in tissue context, mechanical environment, cellular competence, and chromatin state (Vorisek et al., 2022; Li et al., 2019).

### 2.2 Notch functions as a regional, dosage-sensitive, and mechanosensitive gatekeeper

Notch signaling provides another example of regional licensing rather than simple pathway activation. In the AVC and OFT, DLL4–Notch1 signaling is required for EndMT initiation. Genetic loss of Notch1 reduces Snai1 expression, decreases TGF-β2 signaling, and causes endocardial cushion hypoplasia (Zhou et al., 2022; Timmerman et al., 2004). Importantly, Notch activation can prime endocardial cells toward an EndMT-competent state, but complete mesenchymal transformation requires cooperation with BMP2 and other local cues (Luna-Zurita et al., 2010). This supports a model in which Notch serves as a gatekeeper: it helps determine which endocardial cells are allowed to enter the EndMT trajectory, but it is not sufficient to drive the entire process independently. The regional effect of Notch is reinforced by feedback loops that connect endocardial and myocardial programs. Notch-induced Hey1/2 signaling restricts Bmp2 expression, while BMP2-induced Tbx2 helps suppress chamber-specific identity. In turn, Tbx2 can negatively regulate Hey1/2 activity, forming a feedback mechanism that prevents excessive or spatially inappropriate EndMT (Luna-Zurita et al., 2010; Wang et al., 2021). Notch also suppresses VEGFR2, a known inhibitor of AVC EndMT, thereby further shifting selected endocardial cells toward mesenchymal competence (Luna-Zurita et al., 2010; Timmerman et al., 2004). These findings highlight that Notch does not act as a linear EMT switch; rather, its output depends on dosage, regional feedback, and interaction with myocardial signaling.

Mechanical input adds another level of specificity. Hemodynamic shear stress in the AVC and OFT can activate Notch signaling through mTORC2–PKC-dependent regulation of Notch cleavage and NICD release (Mu et al., 2025). This provides a mechanism by which local flow patterns may help restrict EndMT to specific cardiac regions (Mu et al., 2025). Interactions between Notch and mechanotransductive effectors such as YAP/TAZ may further connect shear stress, cytoskeletal remodeling, enhancer activity, and EndMT-associated transcription (Luna-Zurita et al., 2023). Recent evidence that NICD1 may also act within mitochondria to regulate pyruvate dehydrogenase activity suggests that Notch can couple transcriptional, mechanical, and metabolic inputs during cardiac development (Wang et al., 2024a). Although the broader significance of this noncanonical metabolic role requires further validation, it reinforces the idea that EndMT competence is shaped by multiple layers of regional information.

### 2.3 Wnt/β-catenin signaling reveals anatomical specificity of EndMT licensing

Wnt/β-catenin signaling further illustrates the anatomical specificity of developmental EndMT and cautions against viewing this process as a uniform response to pathway activation. Earlier models often treated Wnt signaling as a general EMT inducer. However, genetic and temporal inhibition studies indicate that canonical Wnt signaling is required for EndMT in the proximal OFT, but is largely dispensable for EndMT in the AVC (Bosada et al., 2016). This anatomical difference is central to the continuum model proposed here: the same pathway can be essential in one EndMT niche but nonessential in another. In mouse models, inducible expression of the Wnt inhibitor Dkk1 before EndMT onset disrupts proximal OFT EndMT, whereas later inhibition has a much weaker effect (Bosada et al., 2016; Liebner et al., 2004). This suggests that Wnt signaling acts early to establish EndMT competence rather than continuously driving mesenchymal conversion. Co-culture rescue experiments further indicate that myocardial Wnt activity in the proximal OFT can instruct neighboring endocardial cells through paracrine mechanisms, pointing to a non-cell-autonomous mode of EndMT licensing (Liebner et al., 2004). These findings distinguish proximal OFT EndMT from AVC EndMT and show that regional myocardial identity is a key determinant of endocardial behavior.

β-catenin also has endocardial cell-autonomous functions. Endothelial-specific β-catenin deletion impairs TGF-β2-induced EndMT and reduces cushion mesenchyme formation, even when canonical SMAD phosphorylation remains intact (Liebner et al., 2004). This indicates that β-catenin does not merely operate upstream or downstream of TGF-β signaling, but may provide a distinct transcriptional or structural input required for endocardial transformation. In the AVC, Notch–Wnt–BMP crosstalk further coordinates myocardial–endocardial communication: endocardial Jagged1–Notch1 signaling induces Wnt4, which stimulates myocardial Bmp2 expression and supports cushion formation (Wang et al., 2013a). Together, these data support a region-specific licensing model in which Wnt signaling contributes to EndMT through both myocardial and endocardial mechanisms, with requirements that differ across the AVC and OFT.

### 2.4 ErbB signaling and myocardial–endocardial communication: supportive context rather than core EndMT trigger

Compared with TGF-β/BMP, Notch, and Wnt signaling, ErbB signaling is better positioned as a regulator of myocardial–endocardial communication and tissue-level morphogenesis rather than a core EndMT trigger. Genetic ablation of ErbB2, ErbB4, or NRG-1 in mice causes severe defects in ventricular trabeculation and cushion development, indicating that NRG-1/ErbB signaling is essential for coordinated cardiac morphogenesis (Lee et al., 1995; Gassmann et al., 1995; Meyer and Birchmeier, 1995). These phenotypes support a role for ErbB signaling in the broader developmental environment that permits endocardial transformation and cushion maturation.

However, the direct role of ErbB signaling in EndMT itself remains less clearly defined than that of Notch, BMP/TGF-β, or Wnt. Therefore, this pathway should be discussed with greater conceptual caution. Rather than presenting ErbB as another canonical EndMT pathway, it may be more accurate to view the NRG-1/ErbB axis as part of the myocardial–endocardial signaling network that shapes endocardial competence, cell survival, proliferation, and tissue architecture. Adult studies showing ErbB2-induced EMT-like plasticity in cardiomyocytes are relevant to cardiac repair, but they represent reparative dedifferentiation or EMT-like behavior rather than developmental EndMT (Aharonov et al., 2020; Shakked et al., 2023). These findings may provide useful conceptual parallels, especially through the ErbB2–YAP mechanotransduction axis, but they should not be directly equated with embryonic endocardial transformation.

### 2.5 Chromatin and RNA-based regulation: modulators with variable evidence strength

**FIGURE 2:**
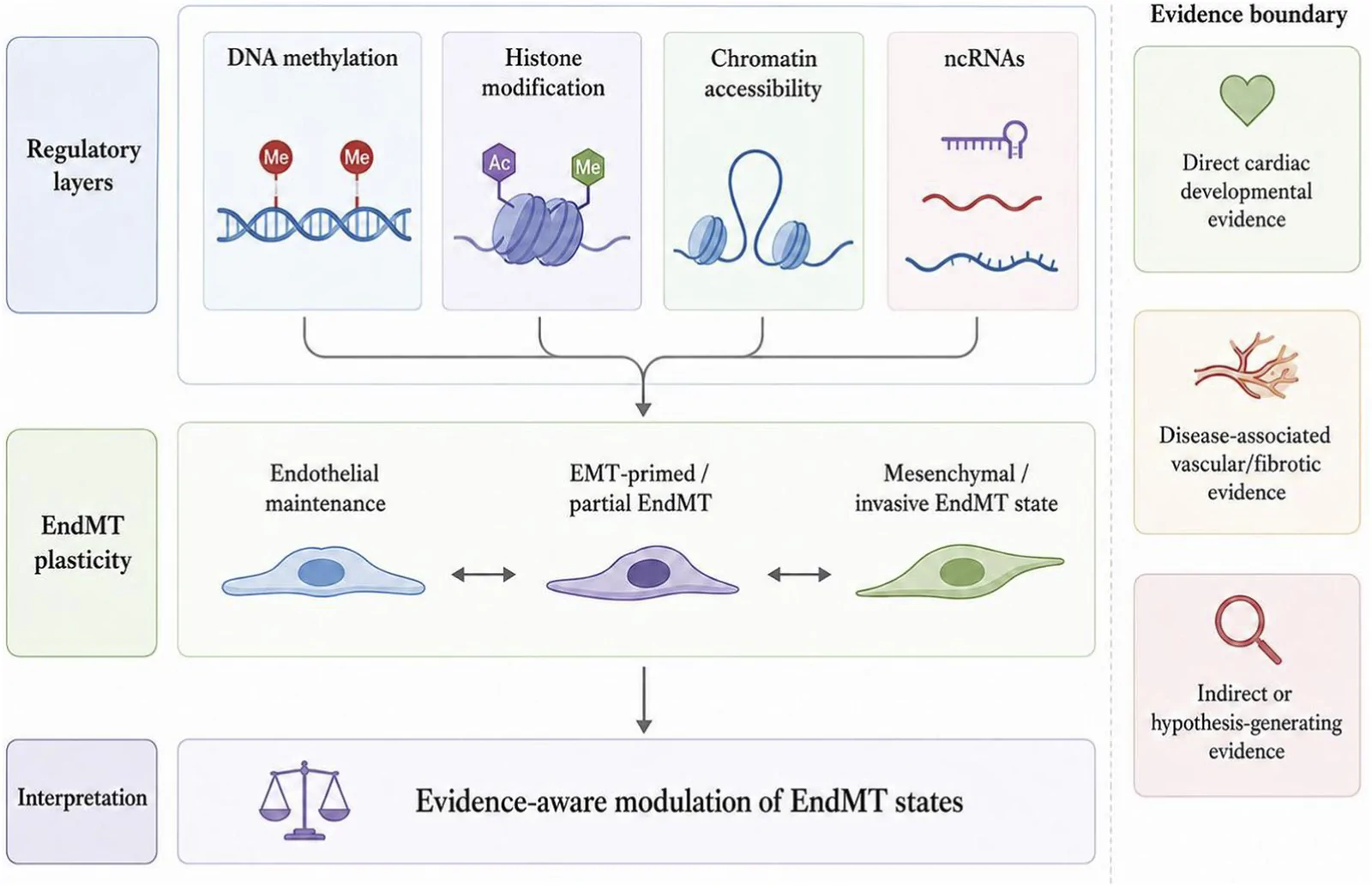
Evidence-aware epigenetic and RNA-mediated regulation of EndMT plasticity. The schematic summarizes major regulatory layers that may tune EndMT competence and transitional cell states, including DNA methylation, histone modification, chromatin accessibility, and noncoding RNA-mediated regulation. These mechanisms can influence endothelial maintenance, EMT-primed or partial EndMT states, and mesenchymal or invasive EndMT states. Because the supporting evidence differs across developmental cardiac, disease-associated vascular or fibrotic, and indirect experimental contexts, epigenetic and RNA-based mechanisms should be interpreted within clear evidentiary boundaries. EndMT, endocardial-to-mesenchymal transition; EMT, epithelial-to-mesenchymal transition; ncRNAs, noncoding RNAs.

Epigenetic and RNA-based mechanisms add another regulatory layer that can modulate the threshold for EndMT licensing, but the strength of causal evidence varies markedly between developmental and adult disease contexts (Figure 2). In developmental EndMT, chromatin regulation appears particularly relevant to the spatial restriction of AVC identity. For example, SMAD4-dependent recruitment of p300 to GATA4-bound enhancers promotes H3K27 acetylation at AVC-associated loci, whereas chamber myocardium uses Hey1/2- and HDAC-associated repression to prevent ectopic activation of AVC-specific enhancers (Stefanovic et al., 2014). This provides direct developmental evidence that chromatin state contributes to regional EndMT competence.

By contrast, many reported roles of DNA methylation, histone modifiers, and non-coding RNAs in EndMT come from adult vascular disease, fibrosis, pulmonary hypertension, cancer, or other non-cardiac systems (Xu et al., 2015; Wang et al., 2026; Liang et al., 2024). These studies are valuable for identifying candidate regulatory modules, but they should be interpreted as indirect or context-dependent evidence when applied to cardiac developmental EndMT. Similarly, miRNAs, lncRNAs, and circRNAs may fine-tune endothelial–mesenchymal plasticity by regulating endothelial identity, mesenchymal gene expression, or TGF-β-associated signaling (Ghosh et al., 2012; Cao et al., 2020a; Yu et al., 2025). However, direct causal links between specific ncRNAs and congenital cardiac EndMT defects remain limited. Therefore, these mechanisms are best considered modulators of EndMT plasticity rather than established universal drivers of developmental EndMT.

Taken together, developmental EndMT is best understood as a regionally licensed continuum of endocardial cell states. Endocardial cells in the AVC and OFT do not undergo mesenchymal transformation simply because individual pathways such as TGF-β/BMP, Notch, Wnt, or ErbB are activated. Instead, EndMT emerges when multiple permissive conditions converge, including regional myocardial signals, endocardial transcriptional competence, chromatin accessibility, extracellular matrix remodeling, and local hemodynamic or mechanical cues. Some endocardial cells remain endothelial, some enter EMT-primed or partial EMT states, and others progress toward invasive cushion mesenchyme. This framework helps reconcile several observations that are difficult to explain using a simple pathway-by-pathway model. Wnt signaling is essential for proximal OFT EndMT but is not equally required in the AVC. Notch activity primes endocardial cells but requires cooperation with BMP and mechanical cues. TGF-β/BMP signaling drives developmental cushion formation in one context but may contribute to pathological mesenchymal activation in another. Epigenetic and ncRNA mechanisms may tune plasticity, but their developmental relevance depends on the strength and origin of supporting evidence. Thus, the key question is not whether a pathway “induces EndMT” in general, but under what developmental stage, anatomical niche, mechanical environment, and cellular state it licenses productive endocardial transformation. To clarify these evidence boundaries, Table 1 summarizes the major mechanisms implicated in developmental EndMT according to species, developmental stage, anatomical region, experimental model, principal findings, unresolved issues, and causal basis.

**TABLE 1:** Comparative evidence for developmental EndMT regulation.

Mechanism/model	Species	Stage/context	Cell type/region	Experimental model	Main finding	Key issue	Causal basis
BMP2–TGF-β2 (Camenisch et al., 2002; Ma et al., 2005; Wang et al., 2005; Song et al., 2007; Bartram et al., 2001)	Mouse; chick	AVC cushion	AVC myocardium/endocardium	Genetic loss; inhibition; cushion assays	Promotes EndMT and cushion mesenchyme	Species- and timing-specific effects	Direct developmental evidence: genetic/perturbation
SMAD4–GATA4–p300 enhancer regulation (Shirai et al., 2009; Rajagopal et al., 2007; Stefanovic et al., 2014)	Mouse	AVC specification	AVC regulatory domains	Chromatin/enhancer studies	Activates AVC EndMT enhancers	OFT and adult relevance unclear	Direct developmental evidence: chromatin/enhancer regulation
Notch1–BMP2 (Luna-Zurita et al., 2010; Timmerman et al., 2004)	Mouse	AVC/OFT development	Endocardial cells	Genetic and explant assays	Notch primes; BMP2 promotes invasion	Notch alone insufficient	Direct developmental evidence: genetic/explant
Hemodynamic Notch (Berg et al., 2025; Mu et al., 2025; Luna-Zurita et al., 2023)	Zebrafish; valve models	Flow-dependent valve/OFT	Shear-stressed endocardium	Imaging; mechanotransduction assays	Flow patterns activate Notch signaling	Full causal chain unresolved	Direct developmental evidence: mechanotransduction
NICD1–PDH (Wang et al., 2024a)	Mouse	Cardiac development	Endocardial cells	Genetic/metabolic assays	Links Notch to metabolism and EndMT	Broader validation needed	Direct developmental evidence: metabolic regulation
Wnt/β-catenin in proximal OFT (Bosada et al., 2016; Liebner et al., 2004)	Mouse	Early OFT	Proximal OFT	Dkk1 inhibition; rescue	Required for OFT EndMT	Not equally required in AVC	Direct developmental evidence: inhibition/rescue
Endothelial β-catenin (Liebner et al., 2004)	Mouse	Cushion formation	Endocardial cells	Endothelial deletion; EndMT assays	Required for TGF-β2-induced EndMT	Mechanism unclear	Direct developmental evidence: cell-specific deletion
Notch–Wnt–BMP relay (Wang et al., 2013a)	Mouse	Early valve development	Endocardial–myocardial interface	Genetic rescue	Coordinates intercellular signaling	Regional generality unclear	Direct developmental evidence: genetic rescue
NRG-1/ErbB (Lee et al., 1995; Gassmann et al., 1995; Meyer and Birchmeier, 1995)	Mouse	Trabeculation/cushion	Myocardial–endocardial interface	Knockout models	Supports morphogenesis	EndMT-specific role indirect	Indirect developmental evidence
Epigenetic/ncRNA (Xu et al., 2015; Wang et al., 2026; Liang et al., 2024; Ghosh et al., 2012; Cao et al., 2020a; Yu et al., 2025)	Mixed cardiac and non-cardiac models	Development and disease	Endothelial/endocardial cells	Omics; *in vitro*; disease models	Modulates EndMT plasticity	Mostly indirect evidence	Hypothesis-generating evidence

Evidence categories are defined as follows: Direct developmental evidence refers to genetic, lineage-tracing, explant, imaging, single-cell, metabolic, mechanotransduction, or functional perturbation studies performed in cardiac developmental contexts that directly interrogate EndMT, or EndMT-related cushion morphogenesis. Indirect developmental evidence refers to developmental cardiac phenotypes or pathway perturbations that support a role in cardiac morphogenesis but do not directly establish EndMT, as the primary mechanism. Disease/repair-related evidence refers to adult fibrosis, valvular disease, myocardial injury, vascular remodeling, pulmonary hypertension, or repair models that reveal EndMT-like plasticity but do not necessarily recapitulate embryonic EndMT. Hypothesis- generating evidence refers to findings derived mainly from adult disease, *in vitro*, omics-based, vascular/fibrotic, or non-cardiac systems that suggest candidate regulatory mechanisms but require further validation in cardiac developmental EndMT. EndMT, endocardial-to-mesenchymal transition; AVC, atrioventricular canal; OFT, outflow tract; BMP, bone morphogenetic protein; TGF-β, transforming growth factor-β; GATA4, GATA, binding protein 4; NICD1, Notch intracellular domain 1; PDH, pyruvate dehydrogenase; NRG-1, neuregulin-1; ErbB, erythroblastic oncogene B receptor family; ncRNA, noncoding RNA.

## 3 Developmental EpiMT: lineage diversification shaped by the epicardial niche

EpiMT provides a second example of cardiac EMT as a context-dependent developmental continuum. Unlike EndMT, which is spatially concentrated in the AVC and OFT endocardium, EpiMT begins with the formation of the epicardial layer and proceeds through epicardial spreading, focal delamination, subepicardial migration, and lineage diversification (von Gise and Pu, 2012). Epicardium-derived cells (EPDCs) arise from proepicardial or epicardial progenitors through species-specific mechanisms (Pérez-Pomares et al., 2002). In zebrafish, proepicardial cells migrate onto the myocardial surface through pericardial fluid movement and active cellular migration (Peralta et al., 2013), whereas in mammals epicardial progenitors arise from foregut mesoderm-derived populations and spread over the developing myocardium under the control of epicardial transcriptional programs and myocardial-derived cues (Cai et al., 2008; Cao et al., 2020b).

In mice, EpiMT occurs after E12.5, following the completion of epicardial coverage, and contributes to coronary vasculogenesis and myocardial growth (von Gise and Pu, 2012). During this window, epicardial cells first establish a continuous epithelial monolayer and then a subset of cells undergoes delamination and invasion into the subepicardial space. These EPDCs contribute predominantly to cardiac fibroblasts, vascular smooth muscle cells, and pericytes, which are essential for coronary vessel maturation, extracellular matrix organization, and myocardial growth. Their direct contribution to coronary endothelial lineages remains more controversial and appears to depend on species, developmental stage, lineage-tracing strategy, and experimental context (von Gise and Pu, 2012; Carmona et al., 2020). Thus, EpiMT should not be interpreted as a uniform conversion of the entire epicardium into mesenchyme, but as a selective and heterogeneous transition in which only specific epicardial subpopulations acquire migratory and lineage-competent states.

### 3.1 Transcriptional competence and lineage bias: WT1, TBX18 and epicardial heterogeneity

A central feature of developmental EpiMT is the establishment of epicardial competence. WT1 and TBX18 are widely used epicardial markers and have been implicated in the control of epithelial integrity, EMT initiation, and EPDC fate specification. Early *in vitro* studies suggested that WT1 and TBX18 exert opposing effects on the EMT regulator SLUG/SNAI2, with WT1 helping to maintain epithelial identity and TBX18 supporting mesenchymal transition under inductive conditions (Takeichi et al., 2013). However, *in vivo* evidence has refined this view. Tbx18 deletion does not markedly impair epicardial formation or early EMT initiation, suggesting that TBX18 is not an essential universal trigger of EpiMT (Greulich et al., 2012). Instead, TBX18 appears to function more prominently at later stages, particularly in EPDC-derived coronary smooth muscle and mural cell differentiation, partly through Notch3- and TGF-β-associated programs (Greulich et al., 2012; Grieskamp et al., 2011).

WT1 has a more consistent role in epicardial activation and EpiMT competence. Loss of Wt1 disrupts transcriptional reprogramming, reduces key signaling components such as Lef1, Ctnnb1, and Raldh2, and impairs EMT and coronary plexus formation (von Gise et al., 2011; Martínez-Estrada et al., 2010). These findings support the idea that WT1 does not simply mark epicardial cells, but helps establish a permissive transcriptional landscape that allows selected cells to respond to Wnt, retinoic acid, and other myocardial-derived signals. Recent single-cell and multi-omic studies of human pluripotent stem cell-derived epicardioids further support this interpretation. Mesothelial epicardial populations co-expressing WT1 and TBX18 can transition toward EMT-primed EPDC states with increased TWIST1 and SNAI2 expression, while retaining heterogeneous lineage potential toward fibroblast, mural, and other mesenchymal-like fates (Meier et al., 2023). This single-cell evidence strengthens the view of EpiMT as a continuum of transcriptional states rather than a discrete switch.

The apparent discrepancy between *in vitro* transcription factor studies and *in vivo* lineage outcomes is informative. Rather than assigning WT1 or TBX18 a single fixed role as either EMT activator or suppressor, it is more appropriate to place them within a competence-and-fate framework. WT1 appears to support the early epicardial state required for EpiMT responsiveness, whereas TBX18 is more closely associated with post-EMT lineage allocation in specific EPDC subsets. This distinction helps explain why marker expression, EMT initiation, and final cell fate cannot be treated as equivalent readouts of EpiMT.

### 3.2 Signaling pathways as niche cues rather than universal EpiMT inducers

Several developmental pathways regulate EpiMT, but their effects depend strongly on the epicardial niche. Rather than acting as isolated EMT inducers, these pathways shape epicardial competence, migratory behavior, and lineage bias in a stage- and context-dependent manner. Wnt/β-catenin signaling is one of the most important pathways for embryonic EpiMT. Epicardial β-catenin activation precedes or accompanies EMT initiation, and disruption of Wnt/β-catenin signaling impairs EPDC mobilization, mesenchymal marker expression, and coronary vascular development (Zamora et al., 2007). However, Wnt activity after adult cardiac injury does not reproduce the full embryonic EpiMT program. Although Wnt reactivation can contribute to epicardial activation and limited cellular mobilization, adult EPDCs usually show restricted regenerative potential and a greater tendency toward fibrogenic outcomes (Duan et al., 2012). This difference emphasizes that Wnt signaling requires an appropriate developmental niche to generate productive EpiMT.

BMP and FGF signaling further illustrate the importance of species, dose, and developmental timing. In avian models, BMP2 promotes proepicardial protrusion formation and myocardial attachment (Schlueter et al., 2006), whereas FGF2 supports epicardial lineage specification and mesenchymal expansion (Kruithof et al., 2006; Mora et al., 2001). However, these signals do not act uniformly across species or experimental models. BMP activity can be dose-sensitive, and excessive BMP signaling may redirect proepicardial cells toward premature cardiomyogenic differentiation *in vitro* (Schlueter et al., 2006). In murine explant models, FGF and BMP ligands alone are insufficient to reproduce robust EpiMT or lineage conversion, suggesting that additional myocardial, mechanical, and extracellular matrix cues are required (Garcia-Padilla et al., 2021; van Wijk et al., 2009). Together, these observations suggest that BMP and FGF signaling should not be viewed through a simple ligand-centered model, but as context-dependent inputs whose effects on EpiMT require a permissive developmental environment.

Notch signaling also acts in a stage- and dosage-dependent manner during epicardial development. Notch2 activity in the proepicardial region helps preserve epicardial progenitor identity by restricting premature myocardial differentiation (Del Monte et al., 2011), whereas Notch1 becomes more relevant in invading EPDCs and contributes to coronary artery development, smooth muscle coverage, and myocardial growth through regulation of lineage differentiation and paracrine signaling (Grieskamp et al., 2011; Del Monte et al., 2011). Notch3 further supports coronary microvascular maturation by promoting pericyte recruitment and mural cell stabilization (Volz et al., 2015). Importantly, excessive Notch activation disrupts epicardial integrity, alters cytoskeletal organization, reduces the subepicardial space, and impairs ventricular development (Del Monte et al., 2011). Thus, Notch does not simply promote EpiMT; it must be precisely tuned to coordinate progenitor maintenance, EMT progression, and vascular lineage maturation.

Taken together, Wnt, BMP/FGF, and Notch signaling should be interpreted as components of a coordinated epicardial niche rather than independent pathway modules. Their effects depend on the developmental stage of the epicardium, the proximity of myocardial-derived signals, the mechanical state of the heart, and the lineage competence of EPDC subsets. This interpretation moves EpiMT away from a pathway catalogue and toward a model in which signaling pathways bias transitional states and cell fates in a context-specific manner.

### 3.3 Mechanical and extracellular matrix cues define the permissive epicardial niche

Biochemical signals alone are insufficient to explain the spatial and temporal behavior of EpiMT. As the embryonic heart begins to contract and expand, the epicardium is exposed to changing mechanical forces, including shear-related movement, tensile stretch, basal traction, and myocardial surface deformation (Andrés-Delgado and Mercader, 2016). These forces influence cell shape, polarity, adhesion, and cytoskeletal organization, all of which are required for epicardial delamination and migration. In mouse models, epicardial deletion of Yap and Taz reduces the expression of EMT regulators such as Snai1, Snai2, and Twist1, impairs epicardial cell invasion, and disrupts coronary vascular formation (Singh et al., 2016). In zebrafish and chick embryos, inhibition of myocardial contraction compromises EPDC invasion, supporting cardiac contractility as an upstream biomechanical driver of EpiMT (Plavicki et al., 2014; Perdios et al., 2019).

The extracellular matrix provides an additional level of niche control. Laminin-α5, hyaluronic acid, fibronectin, and integrin-associated adhesion complexes help support epicardial spreading, basement membrane remodeling, and directional migration (von Gise and Pu, 2012; Craig et al., 2010; Wang et al., 2013b; Dettma et al., 2003). HA–CD44 signaling can activate RhoA/ROCK-mediated cytoskeletal remodeling, while fibronectin and the α4β1 integrin–VCAM1 axis stabilize epicardial attachment and promote migration along the myocardial surface (Allison et al., 2015; Sengbusch et al., 2002). These interactions indicate that ECM molecules are not simply structural substrates; they function as signaling platforms that determine whether epicardial cells remain epithelial, become EMT-primed, or acquire invasive behavior.

Recent work on CCM2 further reinforces the importance of cytoskeletal–ECM coupling. Loss of CCM2 in epicardial cells impairs adhesion, polarity, and migration, disrupts myocardial compaction, and worsens fibrosis after injury (Wang et al., 2024b). This phenotype links developmental EpiMT, myocardial maturation, and adult fibrotic response through a shared requirement for cytoskeletal and matrix organization. However, it also highlights the need to distinguish developmental EpiMT from adult injury-induced epicardial activation. Similar mechanical regulators may be reused in both contexts, but the resulting cellular outcomes differ substantially because the adult injured myocardium provides a stiffer, inflammatory, and less plastic niche.

### 3.4 Developmental EpiMT versus adult EpiMT-like activation

Developmental and adult EpiMT-like programs share molecular features but differ in cellular competence, tissue context, and functional outcome. During embryogenesis, EpiMT occurs within a permissive environment rich in developmental signals, dynamic ECM remodeling, active myocardial growth, and high epicardial plasticity. Under these conditions, EPDCs undergo coordinated delamination, migration, and differentiation into mural, fibroblast, and pericyte lineages that support coronary vasculature formation and myocardial compaction. In contrast, the adult epicardium is largely quiescent under homeostatic conditions and becomes only transiently activated after myocardial injury (Quijada et al., 2020).

After injury, adult epicardial activation is often incomplete and biased toward fibrogenic repair. Lineage-tracing studies have shown that adult Wt1-positive EPDCs contribute mainly to fibroblasts and scar-associated cells rather than robust vascular or myogenic regeneration (van Wijk et al., 2012; Quijada et al., 2019). Adult EPDCs also show reduced motility, lower proliferative capacity, and greater dependence on exogenous stimuli such as TGF-β compared with embryonic epicardial cells (Moerkamp et al., 2016). These findings suggest that adult EpiMT-like activation should not be viewed as a simple reactivation of the embryonic program. Instead, it represents a constrained transitional state shaped by inflammatory signaling, altered matrix stiffness, reduced epigenetic plasticity, and a different metabolic environment.

This distinction is important for translational interpretation. Strategies aimed at enhancing cardiac repair should not merely attempt to activate canonical EpiMT pathways such as Wnt, TGF-β, Notch, or YAP/TAZ. Without restoring the permissive niche that supports productive embryonic EpiMT, pathway activation may instead reinforce fibrogenic remodeling. Therefore, the therapeutic question is not whether EpiMT can be reactivated, but whether adult epicardial cells can be guided away from fibrotic lineage bias and toward reparative vascular or stromal states without impairing myocardial integrity or vascular homeostasis.

The distinction between embryonic EpiMT and adult EpiMT-like activation is summarized in Supplementary Table S1, emphasizing that injury-associated epicardial activation represents a partial and niche-restricted program rather than a full recapitulation of developmental EpiMT.

### 3.5 EpiMT as a niche-dependent lineage continuum

**FIGURE 3:**
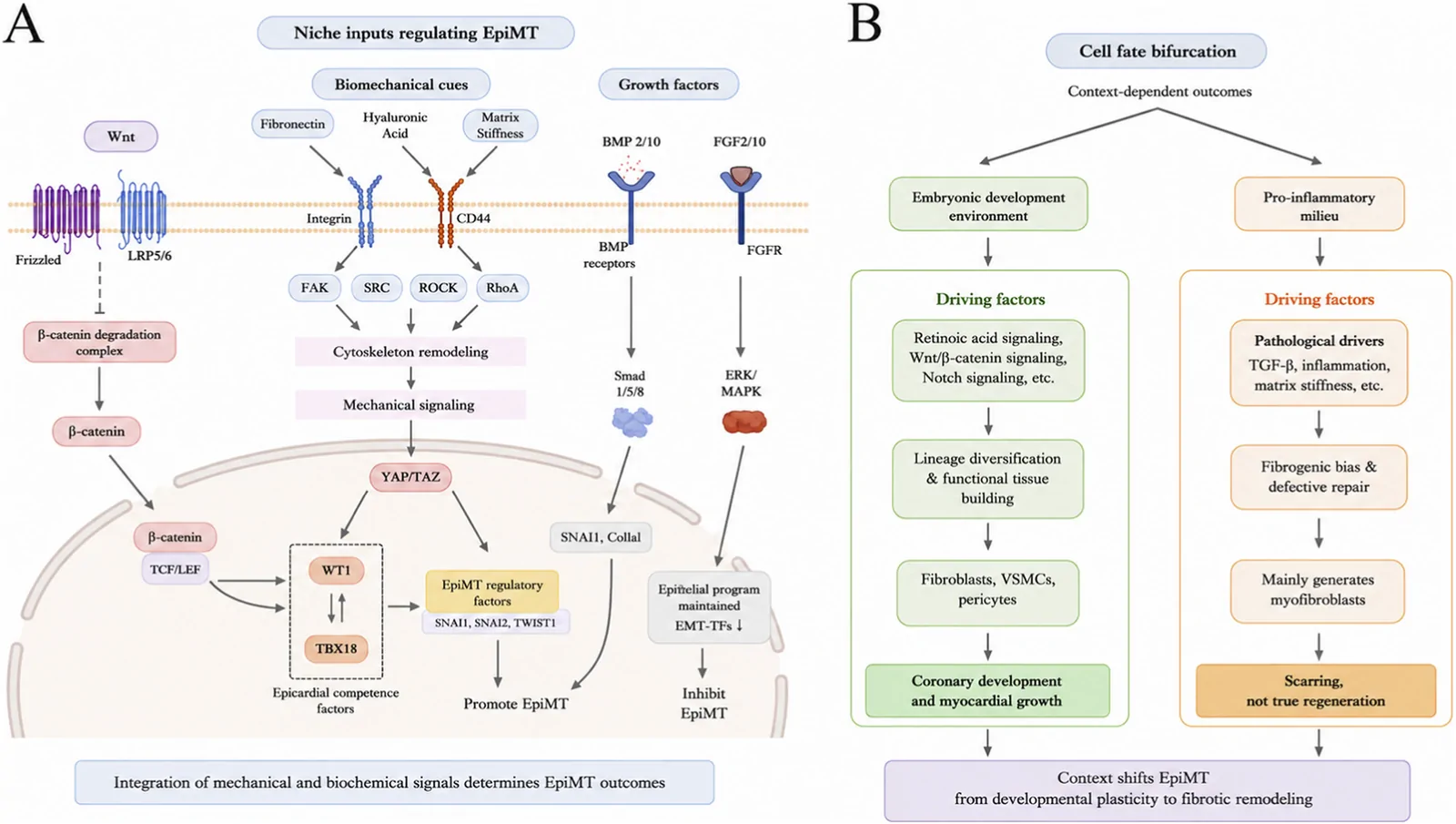
Niche inputs regulating EpiMT and context-dependent cell fate bifurcation. **(A)** Biomechanical and biochemical niche inputs regulating EpiMT. Wnt signaling, extracellular matrix components, matrix stiffness, integrin/CD44-associated mechanotransduction, BMP, FGF, YAP/TAZ, WT1/TBX18-associated competence programs, and EMT-related transcriptional regulators are shown as convergent inputs that influence epicardial plasticity. **(B)** Context-dependent cell fate bifurcation. In the embryonic heart, permissive developmental cues support lineage diversification and functional tissue building, including the generation of fibroblasts, vascular smooth muscle cells, and pericytes that contribute to coronary development and myocardial growth. In a pro-inflammatory or injured environment, similar plasticity-related programs may be redirected toward fibrogenic remodeling, myofibroblast-biased activation, scarring, and limited regenerative output. The figure emphasizes that EpiMT outcomes are determined by niche context rather than by pathway activation alone. EpiMT, epicardial epithelial-to-mesenchymal transition; EMT, epithelial-to-mesenchymal transition; BMP, bone morphogenetic protein; FGF, fibroblast growth factor; YAP, Yes-associated protein; TAZ, transcriptional coactivator with PDZ-binding motif; WT1, Wilms tumor 1; TBX18, T-box transcription factor 18; VSMCs, vascular smooth muscle cells.

Overall, developmental EpiMT is best understood as a niche-dependent lineage continuum (Figure 3). Epicardial cells do not undergo a uniform transition into a single mesenchymal fate. Instead, selected epicardial subpopulations move through epithelial maintenance, EMT-primed or partial EMT states, delaminating and migratory EPDC states, and post-EMT lineage allocation toward lineage-biased derivatives. WT1, TBX18, Wnt, BMP/FGF, Notch, YAP/TAZ, and ECM-associated signals contribute to this process, but none of them acts as an isolated universal driver. Their outputs depend on developmental timing, species, myocardial signaling, mechanical environment, ECM architecture, and the pre-existing competence of epicardial subpopulations (Sanchez-Fernandez et al., 2023; Knight-Schrijver et al., 2022).

This framework also helps reconcile unresolved issues in the field. TBX18 can mark epicardial populations without being indispensable for early EpiMT (Greulich et al., 2012). WT1 is central to epicardial competence but does not define a single final fate (Zhou et al., 2008). Wnt and Notch can support developmental EpiMT, yet their reactivation in adult injury does not automatically restore embryonic regenerative potential (Duan et al., 2012). Mechanical and ECM cues can enable epicardial invasion during development, but in the adult injured heart similar pathways may operate within a fibrotic and restrictive niche (Zeng et al., 2026). Thus, EpiMT should be discussed not as a pathway-driven binary EMT event, but as a context-dependent process in which the epicardial niche governs the extent, direction, and functional outcome of cellular plasticity.

By reframing EpiMT as a niche-dependent lineage continuum, this section provides a conceptual complement to the regionally licensed continuum model proposed for developmental EndMT. In both cases, the key question is not simply which pathways are activated, but when, where, and in which cellular state these pathways permit productive EMT-related transitions and lineage allocation. To avoid treating EpiMT as a uniform pathway-driven event, Table 2 summarizes the evidence for developmental EpiMT regulation and lineage outcomes across species, developmental contexts, cell states, experimental models, and causal evidence.

**TABLE 2:** Comparative evidence for developmental EpiMT regulation and lineage outcomes.

Mechanism/model	Species	Stage/context	Cell type/region	Experimental model	Main finding	Key issue	Causal basis
Epicardial origin/spreading (Pérez-Pomares et al., 2002; Peralta et al., 2013; Cai et al., 2008; Cao et al., 2020b)	Zebrafish; mouse	Epicardial formation	Proepicardial/epicardial cells	Imaging; lineage analysis	Epicardium forms via species-specific mechanisms	Cross-species differences	Direct developmental evidence: imaging/lineage analysis
Developmental EpiMT timing (von Gise and Pu, 2012; Pérez-Pomares et al., 2002; Carmona et al., 2020)	Mouse	E10.5–E12.5	Epicardial cells/EPDCs	Lineage tracing; histology	Subsets undergo delamination and invasion	Not a uniform epicardial transition	Direct developmental evidence: lineage tracing/histology
EPDC fibroblast/mural fates (von Gise and Pu, 2012; Carmona et al., 2020; Volz et al., 2015)	Mouse	Coronary development	EPDC-derived mesenchyme	Fate mapping	Generates fibroblasts, VSMCs, and pericytes	Relative contribution varies by tracing strategy	Direct developmental evidence: fate mapping
EPDC endothelial contribution (von Gise and Pu, 2012; Carmona et al., 2020)	Mouse; zebrafish	Coronary vasculature	Putative endothelial EPDCs	Lineage tracing	Endothelial contribution remains debated	Marker, timing, and species differences	Conflicting developmental lineage-tracing evidence
WT1 competence (von Gise et al., 2011; Martínez-Estrada et al., 2010; Meier et al., 2023)	Mouse; epicardioids	EpiMT initiation	WT1^+^ epicardial cells	Genetic loss; single-cell studies	Supports epicardial competence and activation	WT1 does not define final fate	Direct developmental evidence: genetic/single-cell
TBX18 lineage bias (Takeichi et al., 2013; Greulich et al., 2012; Grieskamp et al., 2011)	Mouse; *in vitro*	Early EpiMT/EPDC differentiation	TBX18^+^ epicardial cells	Deletion; *in vitro* assays	More linked to mural lineage allocation	Early EpiMT role context-dependent	Context-dependent developmental evidence: lineage bias
Wnt/β-catenin (Zamora et al., 2007; Duan et al., 2012)	Mouse; injury models	Embryonic EpiMT/adult activation	Epicardial cells/EPDCs	Conditional perturbation; injury models	Supports embryonic EPDC mobilization	Adult reactivation is incomplete	Direct developmental evidence with disease/repair-related evidence
BMP/FGF signaling (Schlueter et al., 2006; Kruithof et al., 2006; Mora et al., 2001; Garcia-Padilla et al., 2021; van Wijk et al., 2009)	Chick; mouse explants	Proepicardial/early EpiMT	Proepicardial and epicardial cells	Explants; pathway inhibition	Regulates attachment, specification, and expansion	Species-, dose-, and model-dependent	Direct developmental evidence: explant/pathway perturbation
Notch signaling (Grieskamp et al., 2011; Del Monte et al., 2011; Volz et al., 2015)	Mouse	Proepicardium/EPDC invasion	Epicardial progenitors/EPDCs	Conditional models; gain-of-function	Controls progenitor state and vascular maturation	Stage- and dosage-dependent effects	Direct developmental evidence: genetic/gain-of-function
YAP/TAZ mechanotransduction (Andrés-Delgado and Mercader, 2016; Singh et al., 2016; Plavicki et al., 2014; Perdios et al., 2019)	Mouse; zebrafish; chick	Epicardial invasion	Epicardial cells/EPDCs	Yap/Taz deletion; contraction inhibition	Supports EMT regulators and invasion	Interaction with niche signals unresolved	Direct developmental evidence: genetic/mechanotransduction
ECM/integrin cues (von Gise and Pu, 2012; Craig et al., 2010; Wang et al., 2013b; Dettma et al., 2003; Allison et al., 2015; Sengbusch et al., 2002; Wang et al., 2024b)	Mouse; chick; injury models	EpiMT and migration	Epicardium–myocardium interface	ECM and adhesion assays	Supports spreading, adhesion, and migration	Developmental versus injury outcomes differ	Direct developmental evidence with disease/repair-related evidence
Adult EpiMT-like activation (Quijada et al., 2020; van Wijk et al., 2012; Quijada et al., 2019; Moerkamp et al., 2016; Foglio et al., 2024)	Mouse injury models	Post-injury repair	Adult epicardial cells	Injury lineage tracing	Reactivates partial EMT-like programs after injury	Fibrogenic bias; limited regeneration	Disease/repair-related evidence

Evidence categories are defined as follows: Direct developmental evidence refers to lineage-tracing, fate-mapping, genetic, explant, imaging, single-cell, mechanotransduction, extracellular matrix, or functional perturbation studies performed in cardiac developmental contexts that directly interrogate EpiMT, EPDC, migration, or epicardial lineage allocation. Context-dependent developmental evidence refers to developmental studies in which a mechanism affects epicardial competence or EPDC, lineage bias, but its role in early EpiMT, initiation or final lineage outcome varies by developmental stage, cell state, species, or experimental model. Conflicting developmental lineage-tracing evidence refers to lineage-tracing results that differ across species, developmental windows, marker systems, or tracing strategies. Disease/repair-related evidence refers to adult injury, fibrosis, regeneration, or repair studies that reveal epicardial activation or EpiMT-like plasticity but do not necessarily recapitulate embryonic EpiMT. Hypothesis-generating evidence refers to findings derived mainly from adult injury, *in vitro*, omics-based, non-developmental, or non-cardiac systems that suggest candidate regulatory mechanisms but require further developmental validation. EpiMT, epicardial epithelial-to-mesenchymal transition; EMT, epithelial-to-mesenchymal transition; EPDCs, epicardium-derived cells; VSMCs, vascular smooth muscle cells; WT1, Wilms tumor 1; TBX18, T-box transcription factor 18; BMP, bone morphogenetic protein; FGF, fibroblast growth factor; YAP, Yes-associated protein; TAZ, transcriptional coactivator with PDZ-binding motif; ECM, extracellular matrix.

## 4 Aberrant cardiac EMT in congenital heart disease

The regional licensing model of EndMT and the niche-dependent model of EpiMT provide a developmental lens through which congenital heart disease (CHD) can be reconsidered. EMT-related malformations are unlikely to arise simply from excessive or insufficient activation of a single pathway. More often, they may result from a failure to coordinate cellular competence, anatomical location, developmental timing, and lineage allocation. In this view, disease emerges when transitional programs occur in the wrong tissue context, fail to proceed within the proper developmental window, or generate derivatives that cannot support normal cardiac morphogenesis (Berg et al., 2025).

### 4.1 EndMT dysregulation in valve, septal, and OFT defects

Developmental EndMT is largely confined to the AVC and OFT, making these regions particularly sensitive to disruption of the local cues that permit endocardial transformation (Mu et al., 2025; Yang et al., 2025). Evidence from developmental models and CHD-associated variants supports the idea that defective EndMT licensing produces anatomically selective, rather than generalized, cardiac malformations (Berg et al., 2025; Yang et al., 2025). Atrioventricular septal defects and valve malformations are closely associated with impaired cushion cellularization and maturation. In the AVC, BMP/TGF-β–SMAD signaling cooperates with transcriptional regulators such as GATA4 to activate EndMT-associated genes, including Has2, thereby supporting extracellular matrix production and cushion expansion. Perturbation of this regulatory module can reduce mesenchymal cell formation and compromise atrioventricular cushion development, linking defective EndMT competence to septal and valvular abnormalities (Song et al., 2011; Hong et al., 2021).

Semilunar valve defects, including bicuspid aortic valve, further highlight the regional vulnerability of EndMT-dependent structures (MacGrogan et al., 2016). NOTCH1-associated abnormalities can impair endocardial EMT priming, attenuate downstream SNAI1- and TGF-β2-related programs, and disturb valve leaflet maturation (Riley et al., 2011; Kostina et al., 2016). Because Notch output is shaped by dosage, hemodynamic context, and cooperation with BMP and other local cues, Notch-related malformations are best understood as failures of context-specific EndMT licensing rather than as the consequence of a simple linear pathway defect (MacGrogan et al., 2016).

OFT malformations represent another setting in which multiple licensing signals converge within a restricted developmental niche. Wnt/β-catenin signaling is required for proximal OFT EndMT but is not equally required in AVC EndMT, indicating that disruption of the same pathway may produce region-specific rather than global EndMT defects (Bosada et al., 2016). Notch signaling may further connect OFT EndMT with mechanical and metabolic regulation, including shear-responsive NICD activation and the proposed mitochondrial role of NICD1 during developmental EndMT (Mu et al., 2025; Wang et al., 2024a). Whether these noncanonical mechanisms contribute directly to human OFT defects remains to be determined.

EndMT-related malformations may also arise indirectly through altered myocardial–endocardial communication. In developmental models, disruption of NRG1/ErbB signaling impairs trabeculation, cushion development, and tissue-level morphogenesis (Grego-Bessa et al., 2023). These phenotypes are likely to involve defective myocardial–endocardial signaling and altered tissue architecture that secondarily compromise the environment required for cushion maturation, rather than a direct blockade of EndMT initiation.

### 4.2 EpiMT dysregulation in coronary, myocardial, and stromal defects

EpiMT-related CHD phenotypes arise through a distinct but complementary mechanism. Developmental EpiMT generates EPDC populations that contribute to coronary mural cells, fibroblasts, pericytes, extracellular matrix organization, and myocardial growth. Defects in epicardial competence, EPDC migration, or lineage allocation can therefore compromise coronary vascular development, stromal organization, and ventricular wall formation (Ruiz-Villalba and Pérez-Pomares, 2012).

WT1 dysfunction exemplifies impaired epicardial competence. Loss of Wt1 disrupts epicardial transcriptional programs, reduces Lef1, Ctnnb1, and Raldh2 expression, and compromises EPDC mobilization and coronary plexus formation (von Gise et al., 2011; Ramiro-Pareta et al., 2023). These findings suggest that failure to establish a permissive epicardial state can produce combined coronary and myocardial phenotypes, rather than a defect attributable to a single downstream signaling pathway.

Post-EMT lineage allocation represents another vulnerable step. Tbx18 loss does not necessarily prevent early EpiMT, but it can affect later differentiation of EPDC-derived mural or smooth muscle lineages (Wu et al., 2013). TCF21 has also been associated with EPDC-derived fibroblast specification, and its dysregulation may disturb stromal organization and extracellular matrix deposition during heart development (Acharya et al., 2012). These examples underscore an important distinction: entry into an EMT-like trajectory does not guarantee formation of the appropriate derivative lineage.

Epicardial mechanotransduction and ECM–cytoskeletal coupling are also essential for myocardial growth and ventricular wall development. Epicardial deletion of Yap and Taz reduces the expression of EMT regulators such as Snai1, Snai2, and Twist1, impairs EPDC invasion, and compromises coronary vascular development and myocardial growth (Singh et al., 2016). Similarly, loss of CCM2 in epicardial cells disrupts adhesion, polarity, and migration, linking defective epicardial niche organization to impaired myocardial compaction and later fibrotic susceptibility (Wang et al., 2024b). These observations emphasize that EpiMT-dependent morphogenesis depends not only on transcriptional programs, but also on the mechanical and extracellular environment in which epicardial cells become migratory and lineage competent.

### 4.3 Interconnected EMT defects in complex CHD

EndMT- and EpiMT-related defects should not be viewed as isolated events. During heart morphogenesis, the endocardial, myocardial, and epicardial compartments continuously exchange mechanical, extracellular, and paracrine cues (Lavine et al., 2005; Bornhorst et al., 2019). Defective EndMT can alter myocardial growth, local hemodynamics, and extracellular matrix composition, whereas impaired EpiMT can compromise coronary vascular maturation, stromal support, and ventricular compaction (Feulner et al., 2022; Foglio et al., 2024). Complex CHD phenotypes may therefore arise from combined disruption of several EMT-related programs rather than from failure of a single transition.

A key challenge is to distinguish primary EMT defects from secondary consequences of broader morphogenetic disruption. Addressing this issue will require studies that connect CHD genotypes with EMT-related cell-state trajectories, developmental windows, and lineage outcomes. To integrate these developmental and disease-related perspectives, Table 3 compares EndMT and EpiMT in terms of developmental origin, timing, anatomical niche, regulatory principles and mechanisms, downstream cell fates, morphogenetic outputs, and associated CHD phenotypes.

**TABLE 3:** Comparison of developmental EndMT and EpiMT in cardiac morphogenesis and congenital heart disease.

Feature	EndMT	EpiMT
Developmental origin	Endocardial cells	Proepicardial/epicardial cells
Main developmental window	Early cushion formation (∼E9.5 in mouse)	Epicardial activation and EPDC generation (∼E10.5–E12.5 in mouse)
Principal anatomical niche	AVC and OFT endocardium	Epicardium and subepicardial space
Core biological function	Cushion mesenchyme formation for valves, septa, and OFT remodeling	EPDC generation for coronary maturation, stromal support, and myocardial growth
Typical transitional states	Endocardium → EMT-primed → cushion mesenchyme	Epicardium → EMT-primed → EPDCs
Major signaling pathways/inputs	TGF-β/BMP, Notch, Wnt, hemodynamic cues	WT1-related programs, Wnt, BMP/FGF, Notch, YAP/TAZ
Key regulatory principle	Region-specific licensing	Niche-dependent progression and lineage allocation
Major downstream cell fates	Cushion mesenchymal cells	Fibroblasts, VSMCs, pericytes
Main developmental outputs	Valve formation, septation, OFT remodeling	Coronary maturation, myocardial growth, ventricular development
Representative disease associations	Valve defects, AVSD, VSD, BAV, OFT defects	Coronary defects, ventricular hypoplasia, compaction defects
Common regulatory features	Stage-dependent, signaling integration, mechanical sensitivity	Stage-dependent, signaling integration, mechanical sensitivity
Distinctive regulatory features	Restricted to AVC/OFT; cushion-specific cues	Dependent on epicardial competence and EPDC migration
Major unresolved issue	How regional licensing differences (AVC vs. OFT) translate into region-specific CHD phenotypes	EPDC lineage variability and adult reactivation

EndMT, endocardial-to-mesenchymal transition; EpiMT, epicardial epithelial-to-mesenchymal transition; EPDCs, epicardium-derived cells; AVC, atrioventricular canal; OFT, outflow tract; VSMCs, vascular smooth muscle cells; CHD, congenital heart disease; AVSD, atrioventricular septal defect; VSD, ventricular septal defect; BAV, bicuspid aortic valve.

## 5 Evidence boundaries and emerging tools for resolving cardiac EMT states

**FIGURE 4:**
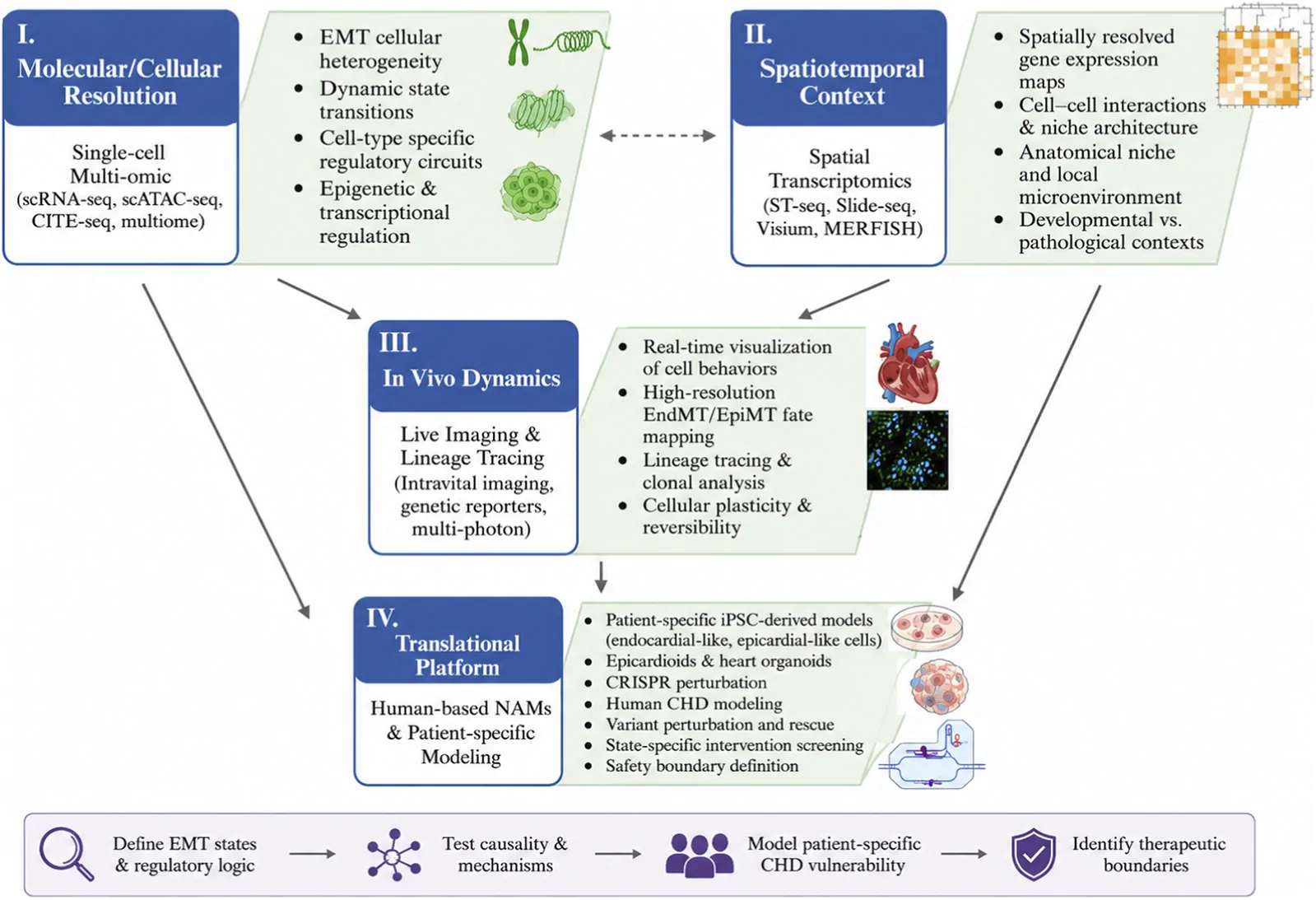
Emerging technologies and human-based NAMs for defining cardiac EMT states and therapeutic boundaries. Single-cell multi-omics can resolve EMT-related cellular heterogeneity, dynamic state transitions, cell-type-specific regulatory circuits, and epigenetic or transcriptional regulation, whereas spatial transcriptomics places these states within anatomical niches and local microenvironments. Live imaging and lineage tracing enable direct assessment of cell behavior, fate mapping, clonal dynamics, plasticity, and reversibility. Human-based new alternative methodologies (NAMs), including patient-specific iPSC-derived endocardial-like and epicardial-like cells, epicardioids, heart organoids, and CRISPR-based perturbation systems, provide platforms for modeling CHD-associated EMT vulnerability, testing candidate mechanisms, and screening state-specific interventions. Together, these approaches can define EMT states and regulatory logic, test causality, model patient-specific disease vulnerability, and identify therapeutic boundaries. EMT, epithelial-to-mesenchymal transition; NAMs, new alternative methodologies; iPSC, induced pluripotent stem cell; CRISPR, clustered regularly interspaced short palindromic repeats; CHD, congenital heart disease.

Because EndMT and EpiMT have been studied across embryonic development, adult disease, injury repair, vascular remodeling, *in vitro* models, and non-cardiac EMT systems, the mechanisms discussed in this review should not be interpreted as having equivalent evidentiary strength. We therefore distinguish three broad levels of evidence. The first is direct cardiac developmental evidence, including embryonic mouse, chick, zebrafish, lineage-tracing, conditional genetic, cardiac explant, and developmental single-cell studies that directly interrogate EndMT or EpiMT during heart morphogenesis. The second is disease-associated or repair-related evidence, including adult fibrosis, valvular disease, myocardial injury, regeneration, vascular remodeling, and pulmonary hypertension models. These studies may reveal EMT-like reactivation or endothelial/epicardial plasticity, but they do not necessarily recapitulate embryonic EndMT or EpiMT. The third is indirect or speculative evidence derived from cancer EMT, general EMT models, non-cardiac epigenetic studies, or ncRNA-based regulatory networks. These findings may identify candidate regulatory modules, but they should be treated as hypothesis-generating rather than definitive evidence for cardiac developmental EndMT or EpiMT. This evidence-aware framework is particularly important for interpreting emerging technologies. Single-cell multi-omics, spatial transcriptomics, lineage tracing, live imaging, organoid models, and AI-assisted analysis should not be viewed as independent topics that broaden the scope of cardiac EMT. Rather, their value lies in helping define which EMT-related states occur *in vivo*, where they occur, whether they represent developmental EndMT/EpiMT or adult EMT-like activation, and how strongly specific mechanisms are supported in cardiac contexts (Figure 4).

Single-cell multi-omics has begun to resolve the heterogeneity of cardiac EMT by identifying transitional endocardial and epicardial cell states that cannot be fully captured by binary marker-based definitions (Lotto et al., 2023; Meier et al., 2023). scRNA-seq can distinguish endothelial or epithelial maintenance, EMT-primed states, migratory mesenchymal populations, and lineage-biased derivatives, whereas scATAC-seq and multimodal approaches can link these transcriptional states to chromatin accessibility and regulatory potential (Meier et al., 2023). In the context of the present review, these approaches are most useful not simply because they generate high-resolution atlases, but because they can test whether proposed EMT continua are supported by ordered cell-state trajectories in defined cardiac regions such as the AVC, OFT, and epicardium (Lotto et al., 2023; Meier et al., 2023; Leshem et al., 2026). Spatial transcriptomics provides a complementary layer by preserving anatomical context. This is essential because both EndMT and EpiMT are spatially restricted processes. In developmental EndMT, spatial profiling may help define how endocardial populations in the AVC and OFT differ from chamber endocardium in signaling response, extracellular matrix interaction, and mechanical sensitivity. In EpiMT, spatial approaches may clarify how epicardial cells transition from an epithelial monolayer to delaminating EPDCs and lineage-biased derivatives within the subepicardial and myocardial environment (Leshem et al., 2026; Mantri et al., 2021; Lázár et al., 2025). Thus, spatial technologies are particularly suited to testing the stage- and niche-dependent framework proposed in this review.

Lineage tracing and live imaging remain essential for distinguishing true lineage transitions from transient marker expression. Cre/loxP-based lineage tracing in mice can define the developmental fates of endocardial- and epicardial-derived cells across embryonic and postnatal stages, whereas genetic labeling and imaging approaches in zebrafish enable analysis of epicardial activation, EMT, migration, and lineage output following cardiac injury (Pollitt et al., 2024; Xia et al., 2022). These approaches are important because EMT marker expression alone cannot establish whether a cell has undergone stable mesenchymal conversion, partial EMT, reversible activation, or lineage diversification. In this sense, lineage tracing and live imaging provide a necessary functional anchor for interpreting single-cell and spatial datasets.

Human-based new alternative methodologies (NAMs), particularly patient-specific induced pluripotent stem cell (iPSC)-derived cardiac cells, iPSC-derived endocardial-like and epicardial-like models, epicardioids, and multicellular heart organoids, are becoming increasingly important for studying cardiac EMT in a human developmental context. These systems do not replace animal models, which remain indispensable for assessing *in vivo* physiology, hemodynamics, tissue integration, and long-range morphogenetic interactions. Rather, they provide complementary platforms in which human genetic background, signaling environment, extracellular matrix composition, metabolic state, and mechanical inputs can be manipulated under defined conditions. For cardiac EMT research, their main value lies in modeling patient-specific developmental vulnerability: iPSC-derived endocardial-like and epicardial-like cells may be used to examine how CHD-associated variants alter EndMT or EpiMT competence, whereas epicardioids and multicellular heart organoids can partially recreate interactions among myocardial, endocardial, and epicardial compartments (Meier et al., 2023; Thomas et al., 2022; Liu et al., 2024). When combined with CRISPR-based genome editing, lineage recording, single-cell multi-omics, and spatial transcriptomics, these models may help test how candidate variants affect EMT-related cell-state transitions, lineage allocation, and tissue organization, while distinguishing primary EMT defects from secondary morphogenetic consequences (Meier et al., 2023; Lv et al., 2025). Such approaches are particularly useful for complex CHD, in which genetic susceptibility, developmental timing, and environmental influences may converge to produce heterogeneous phenotypes (Lv et al., 2025; Schmidt et al., 2023). Nevertheless, current organoid and iPSC-derived systems can approximate only selected features of EndMT or EpiMT and do not fully reproduce embryonic hemodynamics, mature chamber architecture, immune–vascular interactions, placental influences, or the full temporal sequence of *in vivo* heart development (Thomas et al., 2022). Their strongest contribution is therefore not to serve as complete substitutes for embryonic models, but to bridge animal studies and human disease by enabling controlled, patient-specific, and mechanistically testable studies of cardiac EMT, thereby helping to define disease-relevant EMT states and inform precision medicine strategies for congenital heart disease (Thomas et al., 2022; Lv et al., 2025).

AI-assisted image analysis and computational modeling may further improve the quantification of EMT-related states, particularly when combined with live imaging, spatial transcriptomics, and single-cell multi-omics (Wiggins et al., 2023). However, AI should not be presented as an independent mechanistic advance or as a direct route to therapy. Its value lies in state mapping, trajectory inference, feature extraction, and hypothesis prioritization (Zhou et al., 2025). Computational predictions must ultimately be tested using lineage tracing, perturbation experiments, and functional assays.

These evidence boundaries are equally important for interpreting the therapeutic implications of cardiac EMT. Although EMT-related pathways offer potential entry points for intervention, EndMT and EpiMT should not be approached as programs that can be globally activated or inhibited. The same regulatory modules that contribute to pathological EMT-like remodeling also participate in embryonic morphogenesis, coronary vascular maturation, stromal support, adaptive repair, and myocardial–vascular homeostasis (Foglio et al., 2024; Xia et al., 2022). Broad suppression of these pathways may attenuate fibrogenic endothelial or epicardial plasticity, but it could also impair endothelial integrity, coronary vessel stability, wound repair, or epicardial-derived stromal functions. Conversely, indiscriminate activation of developmental EMT programs may increase cellular plasticity while promoting fibrosis, vascular instability, or maladaptive remodeling in the adult heart (Hall et al., 2024; Tombor et al., 2021).

A more realistic therapeutic concept is selective recalibration of pathological EMT-related states. TGF-β/SMAD modulation may be useful for limiting fibrogenic EndMT-like activation or maladaptive stromal remodeling, but its broad inhibition is unlikely to be safe because of its roles in vascular integrity, immune regulation, and tissue repair. Notch-directed strategies face similar constraints, as Notch dosage influences valve development, vascular maturation, and epicardial lineage allocation. Wnt/β-catenin modulation may help control epicardial activation or reparative plasticity, but inappropriate Wnt activation could reinforce fibrotic or aberrant lineage outcomes (Li et al., 2024). Thus, pathway-directed intervention will require careful definition of the target cell type, disease stage, anatomical context, and desired lineage outcome.

Epigenetic and non-coding RNA-based approaches may provide an additional layer of therapeutic specificity. Chromatin regulators, DNA methylation programs, histone-modifying enzymes, miRNAs, lncRNAs, and circRNAs can tune EMT competence, stabilize transitional states, and influence endothelial or epicardial lineage bias. In principle, such mechanisms could allow more selective modulation of disease-associated plasticity than broad pathway blockade (Shen et al., 2025). However, most of these strategies remain preclinical, and their therapeutic value will depend on cell-type-specific delivery, durable but reversible regulation, minimal off-target effects, and validation in cardiac models that preserve anatomical and lineage context (Foglio et al., 2024).

Accordingly, future translational studies should prioritize EMT-state-specific intervention rather than pathway-wide inhibition or activation. Single-cell and spatial technologies can help identify disease-associated EMT-like states; lineage tracing and functional perturbation can test whether these states are causal; and organoid, epicardioid, genome-editing, epigenome-editing, and patient-specific pluripotent stem cell-derived models may provide platforms for screening candidate interventions. A clinically meaningful EMT-informed strategy will require precise modulation of pathological endothelial or epicardial plasticity while preserving vascular stability, myocardial repair, and normal cardiac tissue organization.

Taken together, emerging technologies should be used to refine evidence strength, define EMT-related cell states, and identify safety boundaries rather than broaden the scope of cardiac EMT indiscriminately. The major unresolved questions are not simply which pathways can induce EMT-associated markers, but which EMT-related states exist *in vivo*, which lineages they generate, how stable or reversible they are, and which mechanisms are directly causal in cardiac developmental or disease contexts. Addressing these questions will be essential for distinguishing developmental EndMT/EpiMT from adult EMT-like activation and for determining whether pathological EMT-related plasticity can be modulated without impairing repair, vascular homeostasis, or normal cardiac remodeling.

## 6 Conclusion

Cardiac EndMT and EpiMT are essential morphogenetic programs that shape valve and septal formation, coronary vascular development, myocardial compaction, and cardiac fibroblast generation. Rather than viewing these processes as binary conversions driven by isolated signaling pathways, this review frames them as stage- and niche-dependent continua of transitional cell states. Developmental EndMT is regionally licensed within the AVC and OFT, whereas developmental EpiMT reflects a niche-dependent lineage continuum shaped by epicardial competence, myocardial-derived cues, mechanical forces, and extracellular matrix architecture. Similar pathways, including TGF-β/BMP, Notch, Wnt/β-catenin, ErbB, YAP/TAZ, metabolic regulators, chromatin modifiers, and ncRNAs, may therefore generate distinct developmental, pathological, or repair-associated outcomes depending on cellular context and evidence strength. Distinguishing direct cardiac developmental evidence from adult disease-related parallels and speculative non-cardiac extrapolations will be essential for clarifying EMT-related mechanisms in congenital heart disease, fibrosis, and cardiac repair. Future studies combining lineage tracing, single-cell and spatial omics, live imaging, organoid models, and functional perturbation should focus on defining EMT-related cell states, lineage trajectories, and context-specific windows in which cardiac EMT plasticity can be safely and precisely modulated.
