# Supplementary material for: Cardiac EndMT and EpiMT as a developmental continuum: integration of mechanical, metabolic, and epigenetic regulation
Source: Front Cell Dev Biol. 2026 Aug 14;14:1892888. doi: 10.3389/fcell.2026.1892888 (PMC13527940; doi:10.3389/fcell.2026.1892888)
Supplement: Supplementary file 1 [file Table1.docx]

**Supplementary Table S1 Developmental EpiMT and adult EpiMT-like activation in cardiac development and injury**

| Feature | Developmental EpiMT | Adult EpiMT-like activation |
| --- | --- | --- |
| Biological context | Embryonic heart development | Adult injury or pathological stress |
| Dominant regulatory inputs | RA, Wnt, FGF, Notch, BMP, ECM remodeling | Inflammation, TGF-β, stiffness, injury signals |
| Cellular transition | Coordinated EpiMT with migratory EPDCs | Partial EMT-like reactivation |
| Major lineage tendency | Fibroblasts, VSMCs, pericytes | Fibrogenic/stromal activation |
| Functional outcome | Coronary maturation and myocardial growth | Scar-associated remodeling; limited regeneration |
| Cellular plasticity | High | Restricted |
| Mechanical/ECM environment | Permissive, dynamic ECM | Stiff, fibrotic ECM |
| Evidence interpretation | Developmental morphogenetic program | Partial reactivation, not full recapitulation |
| Translational implication | Reference for lineage diversification | Target to reduce fibrogenic bias |

EpiMT, epicardial epithelial-to-mesenchymal transition; EPDCs, epicardium-derived cells; ECM, extracellular matrix; RA, retinoic acid; VSMCs, vascular smooth muscle cells.
